# Medical students’ perceptions of their learning environment during a mandatory research project

**DOI:** 10.5116/ijme.59c6.086d

**Published:** 2017-10-20

**Authors:** Riitta Möller, Sari Ponzer, Maria Shoshan

**Affiliations:** 1 Department of Medical Epidemiology and Biostatistics, Karolinska Institutet, Stockholm, Sweden; 2 Department of Clinical Science and Research, Södersjukhuset, Karolinska Institutet, Stockholm, Sweden; 3 Departments of Oncology-Pathology and Medical Epidemiology and Biostatistics, Stockholm, Sweden

**Keywords:** Learning environment, undergraduate medical education, scholarly concentration programs, scholarly projects, students’ research projects

## Abstract

**Objectives:**

To explore medical students´ perceptions of their learning environment during a mandatory 20-week scientific research project.

**Methods:**

This cross-sectional study was conducted between 2011 and 2013. A total of 651 medical students were asked to fill in the Clinical Learning Environment, Supervision, and Nurse Teacher (CLES+T) questionnaire, and 439 (mean age 26 years, range 21-40, 60% females) returned the questionnaire, which corresponds to a response rate of 67%. The Mann-Whitney U test or the Kruskal-Wallis test were used to compare the research environments.

**Results:**

The item My workplace can be regarded as a good learning environment correlated strongly with the item There were sufficient meaningful learning situations (r= 0.71, p<0.001). Overall satisfaction with supervision correlated strongly with the items interaction (r=0.78, p < 0.001), feedback (r=0.76, p<0.001), and a sense of trust (r=0.71, p < 0.001).  Supervisors´ failures to bridge the gap between theory and practice or to explain intended learning outcomes were important negative factors.  Students with basic science or epidemiological projects rated their learning environments higher than did students with clinical projects (χ^2^_(3, N=437)_=20.29, p<0.001).

**Conclusions:**

A good research environment for medical students comprises multiple meaningful learning activities, individual supervision with continuous feedback, and a trustful atmosphere including interactions with the whole staff. Students should be advised that clinical projects might require a higher degree of student independence than basic science projects, which are usually performed in research groups where members work in close collaboration.

## Introduction

It is well known that the learning environments (LE) can influence the students’ abilities to achieve the intended learning outcomes, and may, therefore, have impact on the development of their professional behaviors and attitudes.^1^^-^^3^ The LE includes some factors that may contribute to, or affect, students’ learning, such as the physical locations, teachers, learning activities, social relationships and the culture in which students learn.^1^^,^^2^ It may also include students’ experiences or perceptions of their studies^4^ as the quality of the LE is known to influence students’ engagement and success.^5^ Even the social and emotional interactions in the micro learning environment of the student-supervisor relationship can have an impact on the students’ achievements during education.^6^ Therefore, evaluation of LEs facilitates strategies for creating learning experiences that enable the best possible learning outcomes for the students.

Some studies have investigated medical students’ LEs, albeit mostly with a focus on the clinical training environment.^1^^,^^2^^,^^4^ Clinical placements are considered particularly important for learning the clinical skills and attitudes necessary for future professional practice. Warne et al. studied clinical LEs in nine European countries and found that 42% of approximately 2,000 students were satisfied or very satisfied with their LE.^7^ Pales and colleagues who surveyed five Spanish medical schools reported that half of the 4th year students considered their environment more positive than negative.^8^ Nevertheless, many students also indicated several problems, for example expressing challenges when receiving feedback from their supervisors.^8^ This is supported by several studies that have reported that the quality and continuity of the supervision are essential for a positive experience during clinical training.^1^^,^^9^^-^^11^

By contrast, medical students’ perceptions of research training environments have not been systematically studied, as they are a new type of LE. However, individual research projects (also referred to as scholarly research projects) are becoming increasingly common and often mandatory in modern undergraduate medical education.^12^ While the primary objectives of clinical training include taking correct history and conducting a physical examination, the research training focuses on skills development, such as critical appraisal of scientific reports, formulating research questions, collecting, analyzing, and presenting data.^3^ During such projects students usually remain within one LE for a longer period than during any other course throughout their education.^13^^,^^14^ Supervisors or mentors are required to guide and monitor students with the aim to support the development of a deeper scientific understanding and independence.^15^

The theoretical framework of the present study comprises the socio-cultural learning theory of Communities of Practice (CoP).^16^ This theory regards learning not only as the acquisition of knowledge and skills but also as participation in meaningful activities authentic to the field in question, inclusive of interaction and collaboration with others. Thus, learning is about developing the individuals both professionally and personally. Development requires a communicative interaction between the more knowledgeable other, e.g., supervisor, and the learner which, may occur by instruction, modeling or scaffolding. Scaffolding means a structure of support or process by which the more competent person helps the student to learn a new task while withdrawing the support when it becomes unnecessary. Thus, the student starts as a peripheral participant but moves to a more central position in the community after expanding his or her knowledge and skills in the field.^16^ We, therefore, reasoned that during scientific research projects, CoPs form the core component of students’ LE.

We hypothesized that although today´s research environments may constitute good examples of CoPs, the complexity of modern medical research and aspects of competition may to some degree render them suboptimal as LEs. To initiate analysis and assessment of authentic research environments as LEs, this study aimed to explore medical students´ perceptions of their LEs during a mandatory scientific research project. Therefore, we sought to identify what characteristics of the LE that are perceived as beneficial by the students. To the best of our knowledge, no such studies have been performed in this area.

## Methods

### The context of the study 

Participants were recruited from a medical university with a 5.5-year (11 semesters) medical program. Students were included if they had completed the mandatory research project (20 weeks) during semester 7. After the research project course, students should have a deeper understanding of the scientific basis of medicine, and ability to interpret and evaluate scientific literature to become scientifically proficient clinicians. The students individually plan and carry out a research project, and present a research report essentially formatted as a scientific publication. Supervisors are active researchers with at least a PhD degree who can offer a suitable project in their area of expertise. Optional co-supervisor(s) may, for instance, be PhD students or other researchers in the same area. In addition to the supervisor(s), the progress of each project is monitored by research-active coordinators appointed specifically to ensure that the projects are suitable as student projects according to the university guidelines. Each coordinator is responsible for approximately 10-15 students per semester, he or she arranges three seminars (project plan, half-time, and examination), and acts as a tutor and examining teacher on these occasions. A grading basis of pass or fail is applied. On average 85% of the students pass the examination on the first occasion.

### Study design and participants 

A total of 651 medical students who enrolled in the current study between 2011 and 2013 were approached to participate in a cross-sectional questionnaire-based study. An explanatory statement outlining the aims, methods and voluntary nature of the project was provided to students both verbally and in written format. Project staff did not take part in final grade allocation.  The questionnaires were distributed by email at the end of the project semester but before students had received the final results of the examination. Participation was voluntary, and consent was implicit in returning the questionnaire. In total, 439 students (mean age 26 years, range 21-40, 60% females) returned the questionnaire corresponding to a response rate of 67%. The majority of the students carried out a project in a clinical environment (n=256, 58%) while the rest of the projects were classified as basic science (n=104, 24%), epidemiological (n=53, 12%) or other, e.g. leadership, management, or medical education projects (n=26, 6%). The projects were carried out in 22 out of 23 departments at the university. The study was approved by the Regional Ethical Review Board, Karolinska Institutet. All data were anonymized using unique identification code numbers and were stored in a secure location.

### Data collection methods 

Although there are several questionnaires available for assessing students’ perceptions of their clinical LE, no instrument has been cross-culturally validated in the Swedish context to evaluate students' perceptions of research environments specifically. Therefore, we chose to use the Swedish version of the validated Clinical Learning Environment, Supervision and Nurse Teacher (CLES+T) questionnaire^17^^-^^19^ for this study. CLES+T has shown satisfactory psychometric properties.^14^

The CLES+T consists of 31 items and five sub-dimensions which include: 1) pedagogical atmosphere (9 items), 2) leadership style (4 items), 3) premises of nursing care (4 items), 4) supervisory relationship (8 items), and 5) the role of the teacher (6 items). In our context, the sub-dimensions leadership style and premises of nursing care were irrelevant and were therefore replaced with supervisor, coordinator and student co-operation (3 items). Thus, our modified questionnaire consisted of 36 items and 4 sub-dimensions: 1) pedagogical atmosphere (9 items), 2) supervisory relationship (8 CLES+T supervisory relationship items and 4 of 6 items of the role of the teacher), 3) coordinator relationship (8 CLES+T supervisory relationship items and 4 of 6 items of the role of the teacher) and 4) supervisor, coordinator and student relationship (3 items). The participants were asked to rate the items on a scale from 1 (not at all; disagree) to 5 (totally agree).

### Data analysis 

Descriptive statistics were used to characterize data and to describe the population features. The Mann-Whitney U test was used to compare two independent groups while the Kruskal-Wallis test was used when more than two independent groups were compared. For post-hoc analyzes the Nemenyi test was performed. Bivariate correlation between the sub-dimensions and the statements was calculated with Spearman correlation coefficient. The level of significance was set to 0.05. Bonferroni correction was used for multiple analyzes. The statistical analyzes were performed with R version 3.1.

## Results

The mean scores of the sub-dimensions varied between 3.85 and 4.30 (Table 1). The mean scores for single items (italicized below) within sub-dimensions varied from 3.50 to 4.64. Overall, male students rated all items somewhat higher than female students did, although The supervisor could integrate theoretical knowledge with practical work was the only item for which the difference was statistically significant (U=13899, p<0.001) (data not shown). Overall, there was no correlation between the studied items and the results (pass/fail) in the final examination.

**Table 1:** Mean and standard deviation (SD) for all sub-dimensions

Sub-dimension		Mean (SD)
Pedagogical atmosphere		4.22 (0.83)
	- Clinical projects	4.09 (0.85)
	- Basic science projects	4.46 (0.75)
	- Epidemiological projects	4.43 (0.65)
	- Other^*^	4.06 (0.93)
Supervisory relationship		4.30 (0.88)
	- Clinical projects	4.18 (0.96)
	- Basic science projects	4.45 (0.78)
	- Epidemiological projects	4.55 (0.65)
	- Other^*^	4.38 (0.69)
Coordinator relationship		4.13 (0.96)
	- Clinical projects	4.07 (1.01)
	- Basic science projects	4.29 (0.82)
	- Epidemiological projects	4.34 (0.84)
	- Other^*^	3.66 (1.00)
Supervisor, coordinator and student relationship		3.85 (1.11)
	- Clinical projects	3.79 (1.12)
	- Basic science projects	4.07 (1.04)
	- Epidemiological projects	4.04 (1.02)
	- Other^*^	3.09 (1.08)

*Projects including leadership, management, and medical education

### Pedagogical atmosphere 

The pedagogical atmosphere was considered positive by a majority of the students (Table 2). Accordingly, the staff was found to be easy to approach, and taking part in discussions was not perceived as uncomfortable. The results indicated a strong correlation between the items My workplace can be regarded as a good learning environment and There were sufficient meaningful learning situations (r=0.71, p<0.001). Likewise, a moderate correlation was found between the item My workplace can be regarded as a good learning environment and items pertaining to an encouraging atmosphere. The learning situations were multi-dimensional (r=0.67, p<0.001), I felt comfortable going to my workplace (r=0.66, p<0.001), There was a positive atmosphere at my workplace (r=0.62, p<0.001). The staff was interested in student supervision (r=0.63, p<0.001), and during the meetings I felt comfortable taking part in the discussion (r=0.60, p<0.001). On the other hand, there was no statistically significant correlation between the pedagogical atmosphere and supervisory relationship.

### Supervisory relationship 

A majority (92%) of the students reported their supervisors as showing a fairly positive or very positive attitude towards supervision, and that they had received continuous feedback from their supervisors (Table 3). In line with this, most students (89%) felt they had been given the individual supervision implicit in each project structure, and that a sense of trust characterized the supervisory relationship. Thus, the item Overall I’m satisfied with the supervision I received showed a strong correlation with the items individual supervision (r=0.74, p<0.001), continuous feedback (r=0.76, p<0.001), mutual interaction in the supervisory relationship (r=0.78, p<0.001) and a sense of trust (r=0.71, p<0.001). The lowest scores were given for supervisors’ ability to bridge the gap between the theoretical and practical knowledge and to explain the intended learning outcomes of the research project course.

**Table 2:** Mean and standard deviation (SD) in the sub-dimension Pedagogical atmosphere* (N=439)

Pedagogical atmosphere	Mean (SD)	p-value^**^
The staff was easy to approach	4.35 (0.92)	0.08
I felt comfortable going to my workplace	4.28 (1.04)	0.49
During the meetings I felt comfortable taking part in the discussion	4.32 (0.97)	0.44
There was a positive atmosphere in my working place	4.43 (0.89)	0.17
The staff was generally interested in student supervision	4.23 (1.04)	0.35
The staff got to know the students by their personal names	4.32 (1.11)	< 0.01
There were sufficient meaningful learning situations	3.90 (1.25)	< 0.001
The learning situations were multi-dimensional in terms of content	3.89 (1.20)	< 0.001
My workplace can be regarded as a good learning environment	4.23 (1.07)	< 0.01
Mean score for the sub-dimension	4.22 (0.83)	0.001

*The items were rated on a scale from 1=not at all; disagree to 5 totally agree.

**The differences between students doing basic science projects and clinical projects

**Table 3:** Mean and standard deviation (SD) for sub-dimension Supervisory relationship (N=439)

Supervisory relationship	Mean (SD)	p-value^*^
My supervisor showed a positive attitude towards supervision	4.64 (0.79)	0.71
I felt I received individual supervision	4.56 (0.88)	0.06
I continuously received feedback from my supervisor	4.24 (1.11)	0.08
Overall I’m satisfied with the supervision I received	4.32 (1.11)	0.06
The supervision was based on a relationship of equality and promoted my learning	4.36 (1.07)	0.23
There was a mutual interaction in the supervisory relationship	4.33 (1.05)	0.18
Mutual respect and approval prevailed in the supervisory relationship	4.43 (1.01)	0.17
The supervisory relationship was characterized by a sense of trust	4.35 (1.06)	0.17
The supervisor could integrate theoretical knowledge with practical work	4.43 (0.95)	0.34
Supervisor could clarify the learning outcomes for the research project	3.70 (1.31)	<0.01
Supervisor helped me to bridge the gap between theoretical and practical knowledge	4.07 (1.20)	0.08
Supervisor and the staff collaborated to support my learning	4.11 (1.19)	0.02
Mean score for the sub-dimension	4.30 (0.88)	0.03

*The differences between students doing epidemiological projects and clinical projects

### The impact of the type of research area 

Students who carried out basic science or epidemiological studies rated their LE (χ^2^_(3, N=433)=_21.78, p<0.001), and supervision (χ^2^_(3, N=429)_=13.10, p<0.01) higher than students who carried out studies in a clinical environment did. The following items regarding pedagogical atmosphere were rated higher by the students with basic science projects: There were sufficient meaningful learning situations (χ^2^_(3, N=438)=_27.56, p<0.001), The learning situations were multi-dimensional in terms of content (χ^2^_(3, N=435)_=22.42, p<0.001) and The staff got to know the students by their personal names (χ^2^_(3, N=438)_=18.92, p<0.001). By contrast, students who performed epidemiological studies rated higher the items Supervisor could clarify the learning outcomes for the research project (χ^2^_(3, N=438)_=17.01, p<0.001) and Supervisor and the staff collaborated to support my learning than did students in clinical projects (χ^2^_(3, N=436)_=17.91, p<0.001).

Students with projects in the category Other rated items in the sub-dimension Coordinator relationship lower than other students did, and the difference to students in basic science was statistically significant (χ^2^_(3, N=426)_=12.87, p<0.01). Furthermore, in the sub-dimension Supervisor, coordinator and student relationship, students with projects in the category Other rated the item In our meetings I felt like we were colleagues significantly lower than students in basic science (χ^2^_(3, N=430)=_22.91, p<0.001) did.

## Discussion

This study is the first to explore students’ perceptions of their LE during medical students’ mandatory research projects. It is based on the actual experiences of students who are not a priori specifically interested in research. The study covers 22 different departments or clinics and 439 individual projects in such diverse areas as neurology, cardiology, neonatal care, in vitro basic science and much more. The two most important aspects of our study were the pedagogical atmosphere and supervisory relationship. The main findings were that there was a strong correlation between the items good learning environment and meaningful learning situations. Overall satisfaction with the supervision correlated strongly with the items concerning interaction, feedback, and a sense of trust. Significant findings were also that the students’ experiences of the two most important aspects differed between basic science and clinical projects, and that no correlation between the studied items and the results (pass/fail) in the final examination was found.

### Characteristics of a good learning environment 

Learning in authentic practice is important for the development of students’ understanding of the research process and scientific attitude^5^^,^^6^, in particular for medical students whose focus may otherwise be mainly on clinical skills. Ideally, the research LE should foster academic and personal development as well as encourage collaboration and teamwork. Our results indicate that a good LE is characterized by individual supervision, continuous feedback and a positive atmosphere which includes varied learning situations, interactions, discussions and a sense of trust. Reviewing the literature, we found no other systematic studies of research environments for comparison. However, interviews with previous supervisors^20^ and a literature review^21^ support our findings. MacDougall and Riley recommended one main supervisor, regular meetings and involving the students early in the whole research team in creating optimal circumstances for the supervision of the students.^20^ In a review by Chang and Ramnanan focusing on students’ experiences of research projects, interaction with faculty was considered as an essential factor.^21^ In comparison to results from clinical learning environments^7^^,^^22^ students’ ratings of pedagogical atmosphere and of the supervisor (mentor) relationship seem to be higher in research environments. However, these differences have to be interpreted with caution since clinical placements are shorter and students usually have several supervisors during those periods.

Our results also implied that satisfaction with supervision correlated strongly to a sense of trust in a supervisory relationship. Trust may be crucial in the sense that, as the projects constitute authentic research, the results are unknown beforehand, and students must learn to solve problems in a culture that incorporates doubt and uncertainty as part of the research process.^1^^,^^22^^-^^24^

### Differences between types of environment 

An unanticipated finding was the differences in students’ ratings depending on the type of project. Students in basic science and epidemiological projects gave highest rates for their LE and supervision. One explanation may be that in these settings research is usually carried out in groups where members work in physically as well as temporally close collaboration. Thus, students could become active members of a CoP that provided guidance from several persons/experts, which potentially led to several learning opportunities. The observation that in such LEs the group members learnt and used students’ names may indicate an enhanced feeling of connectedness to the group. By contrast, in clinical environments, the research team seldom works fulltime on the project or on a daily basis in close vicinity to each other. Therefore, the collaboration may have to be scheduled rather than integrated into the daily work schedule.^16^ Thus, it is possible that clinical projects require a higher degree of independence from students than other projects, a topic that should be discussed with the students when they choose their projects. It should also be kept in mind that there are possible cultural differences regarding attitudes to learning and supervision in different environments that may be reflected in students’ answers. As concerns have been expressed about the future lack of clinical and translational scientists, it would be unfortunate if curricular research projects deter the future doctors from research. Unfortunately, there are no published studies for comparison with more research required in this area.

### The role of coordinators

The two items with lowest scores were about interaction with the course coordinators and about supervisors’ abilities to clarify the learning outcomes. By organizing seminars and giving feedback on various stages of the report, the coordinators help students stay on track and to fulfill the requirements of the university. Coordinators also bring a degree of objectivity to students’ experiences and help them reflect on their experiences. Therefore, the coordinator relationship is important but also a possible source of frustration. There are several explanations to why students working with projects within the category Other were least satisfied with this relationship. The projects in this category were heterogeneous and often bordered on non-medical subjects, wherefore it was not always possible to find a coordinator with suitable background knowledge. Moreover, the less satisfied students were often also found among those who went abroad for their projects and were in contact with their coordinator only by e-mail or Skype. Similarly, several factors may explain the lower rating of supervisors’ abilities to clarify the learning outcomes. Although students´ expectations may have been false and/or too high, the results primarily suggest that a clear distinction should be made between outcomes expected by on the one hand the supervisor and on the other hand the university, i.e., between the research goals and the educational goals. We recommend clarifying the roles and expectations to students before they start their projects.

### Learning environment and communities of practice

CoP is a group of people who share the same concern or passion for something they do and learn to do better as they interact regularly.^16^ Mutual engagement characterizes a successful CoP; thus, Wenger saw learning as a social phenomenon.^16^ Our results are not faculty-based evaluations of LEs or student learning but reflect students’ own experiences. Hence, they are in line with the theory of CoP as they emphasize the importance of practical, authentic involvement and social interaction.^16^ This may be of particular importance when aiming to achieve an understanding of science and research, as they are multi-faceted domains. The fact that our study showed a strong correlation between specific items and overall contentment indicates that the students nevertheless perceived not only individual feedback but also interactions and feelings of inclusion as important LE factors. Thus, our findings suggest that student-supervisor interaction and mutual engagement created the scaffold^16^ that was essential in the LE during research projects because both student and supervisor influenced the outcome. When this scaffold grows to include staff and coordinators, it will result in even better LEs for students’ development. However, it must be noted that creating a good research CoP for students requires that sufficient time is allocated for supervision and for students to become members of these communities. Schönrock-Adema et al.^5^ have suggested a theoretical framework according to which the educational environments may be investigated using three social domains: personal development, relationship and system maintenance or system change. In our study we have investigated the essential student-supervisor relationship, as a part of the research organization, but the system dimension5 has not been the focus of the current study.

### Strengths and limitations

The sample size and the diversity of research environments together with highly significant results for many items represent strengths of the study. One minor limitation concerns the questionnaire. Tools have been developed to assess students’ LEs but these have mainly focused on assessing clinical settings and offer limited insight into research environments.^25^ However, the original CLES+T questionnaire is validated in Swedish,^18^ and the modifications were few, and the general design of the original instrument was preserved. In addition, as far as we know there are no validated instruments to evaluate research environments specifically. Secondly, the questionnaire was answered within three weeks after the course, an interval that is probably short enough to exclude memory bias but long enough to allow students to acquire a certain distance to their placement. Lastly, although our data reflects a positive attitude towards LEs and supervision, the non-responders may have been less satisfied with their experiences. Previous research has shown that students with good examination results assess the environment more positively than those with poorer results.^26^^,^^27^ Importantly, our survey was launched at the end of the course but before the examination period and there was no correlation between the tested items and the success in the final examination. Thus, we consider the results reliable since we had a large sample with a sufficient response rate for students included from several semesters to minimize fluctuations between student cohorts. Consequently, we believe that our results are generalizable to other universities.

### Future studies

There is a need for future studies that should carefully examine the local culture of research organizations, the method of supervision, and the student’s role in this culture. Such a study could aim to specify what students identify as central to good supervision and essential to achieving the learning outcomes in a research environment. It would also be interesting to examine how supervisors perceive the environment and how student participation in research influences supervisors and their research groups. Finally, the students’ perceptions of the physical learning spaces during such a long placement as research projects should be explored.

## Conclusions

In conclusion, by providing insight into students’ LEs across several research settings, the current study offers directions for further development of student research programs. The core elements in good research LEs are meaningful learning activities, individual supervision, continuous feedback and a positive atmosphere including interaction with the whole staff. Students should be informed that research environments may differ, with clinical research perhaps demanding a higher degree of independence than basic science or epidemiological studies. The differences between research areas indicate a need for further studies in the micro-LE.

### Acknowledgements

The authors wish to thank Annika Tillander for statistical assistance as well as the medical students at Karolinska Institutet who devoted their time to participate in the study. This work was supported by Karolinska Institutet and Karin and Nils Rosander’s foundation.

### Conflict of Interest

The authors declare that they have no conflict of interest.
